# Mechanisms of Regulating Cell Topology in Proliferating Epithelia: Impact of Division Plane, Mechanical Forces, and Cell Memory

**DOI:** 10.1371/journal.pone.0043108

**Published:** 2012-08-17

**Authors:** Yingzi Li, Hammad Naveed, Sema Kachalo, Lisa X. Xu, Jie Liang

**Affiliations:** 1 School of Biomedical Engineering and Med-X Research Institute, Shanghai Jiao Tong University, Shanghai, China; 2 Department of Bioengineering, University of Illinois at Chicago, Chicago, Illinois, United States of America; 3 Shanghai Center for Systems Biomedicine, Shanghai Jiao Tong University, Ministry of Education, Shanghai, China; University of Reading, United Kingdom

## Abstract

Regulation of cell growth and cell division has a fundamental role in tissue formation, organ development, and cancer progression. Remarkable similarities in the topological distributions were found in a variety of proliferating epithelia in both animals and plants. At the same time, there are species with significantly varied frequency of hexagonal cells. Moreover, local topology has been shown to be disturbed on the boundary between proliferating and quiescent cells, where cells have fewer sides than natural proliferating epithelia. The mechanisms of regulating these topological changes remain poorly understood. In this study, we use a mechanical model to examine the effects of orientation of division plane, differential proliferation, and mechanical forces on animal epithelial cells. We find that regardless of orientation of division plane, our model can reproduce the commonly observed topological distributions of cells in natural proliferating animal epithelia with the consideration of cell rearrangements. In addition, with different schemes of division plane, we are able to generate different frequency of hexagonal cells, which is consistent with experimental observations. In proliferating cells interfacing quiescent cells, our results show that differential proliferation alone is insufficient to reproduce the local changes in cell topology. Rather, increased tension on the boundary, in conjunction with differential proliferation, can reproduce the observed topological changes. We conclude that both division plane orientation and mechanical forces play important roles in cell topology in animal proliferating epithelia. Moreover, cell memory is also essential for generating specific topological distributions.

## Introduction

Regulation of cell growth and cell division plays fundamental roles in tissue formation, organ development, and cancer progression [1]–[6]. Proliferating epithelial monolayer, a two-dimensional sheet of dividing cells that adhere to each other tightly, is an excellent model system widely used to study cell growth and cell division [7], [8]. Studying epithelial system can also lead to understanding other important biological processes such as tumorigenesis [5], [9].

**Figure 1:**
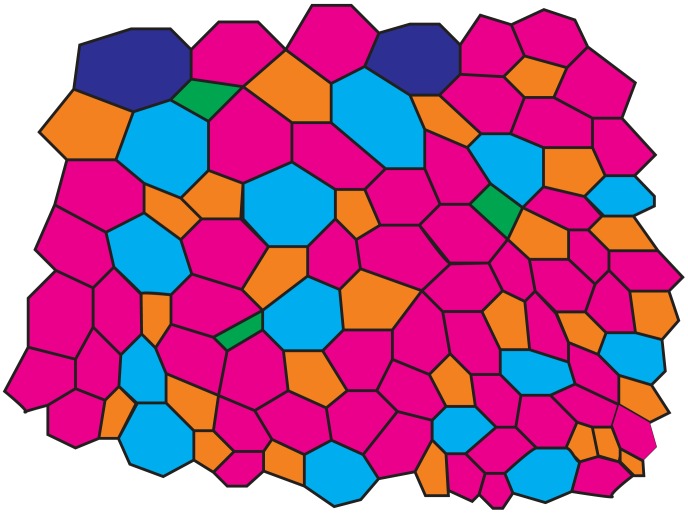
Polygonal representation of cell structure in natural epithelia. An epithelial cell is represented as an 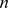-sided polygon depending on the number of neighboring cells it has. This epithelial tissue is composed of 4-sided (green), 5-sided (orange), 6-sided (magenta), 7-sided (light blue), 8-sided (dark blue) cells.

Cell geometry and cell topology are two important aspects when studying cell growth and cell division. Cell geometry refers to the shape and size of a cell, as well as lengths and angles of cell boundaries [7], [10]. Cell topology refers to cell connectivity, which is quantified by the number of cell neighbors [7], [11]. Specifically, a cell with 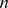 adherent neighboring cells can be regarded as an 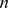-sided polygon (Fig. 1, see [10], [12], [13] for details). Changes in cell topology refer to changes in the number of neighboring cells that a cell contacts, namely, a cell gains or loses neighbors.

Cell geometry and cell topology are tightly connected [14]. For example, the number of cell sides is linearly correlated with cell size [10]. The topological distribution of mitotic cells in *Drosophila* is regulated by an area-dependent growth rate [15]. Cell topology is also modified by dynamic changes in cell-cell contacts, which occur in a variety of biological processes, including cell division, cell rearrangement, and cell death [16]–[19]. Studying geometric properties of cells and the underlying biological processes can provide important insights into the mechanisms of regulating cell topology in proliferating epithelia [20].

The topological structure of proliferating epithelia has been studied both experimentally and theoretically since the early 20th century [14], [21]–[23]. In the 1920s, Lewis observed a skewed distribution of cell polygonal types in the cucumber epidermis. Consistent with Euler's theorem [16], the average number of neighbors of a cell was found to be approximately six due to the prevalent three-cell junctions. The distribution is dominated by hexagonal cells, with a narrow range from four-sided to nine-sided cells [14], [21]. In addition, there are more five-sided cells than seven-sided ones. Subsequent studies showed that topological distributions are strikingly similar in both animals and plants [16], [24], although the molecular architecture of these cells can be quite different [7]. For example, a similar distribution of cell polygonal types is observed with a peak of approximate 45% hexagons in *Cucumis*, *Drosophila*, *Xenopus*, *Hydra*, and *Anagallis* [16]. This remarkable similarity in topological distributions suggests that despite differences in molecular architectures, there exist fundamental mechanisms common to different species which result in this similarity in topological distributions.

At the same time, different mechanisms acting through local properties may produce quantitatively variant distributions among different species. For example, *Anacharis* has a hexagonal cell frequency as high as 57% [24], which is significantly different from the hexagonal frequency of around 45% observed in other species. In addition, localized differential proliferation may also affect the topological distribution of cell polygonal types. Gibson *et al*. showed that when a clone of rapidly proliferating cells are bounded by quiescent cells, the boundary proliferating cells presented a significant shift in the distribution of cell polygonal types with fewer sides than natural proliferating cells [16].

Several computational models have been developed to study the mechanisms of regulating cell topology. They include topological models and mechanical models [8], [11], [15], [16], [22], [25]–[27]. Although these studies have lead to important insight into understanding the regulation of cell topology, there are some limitations with these models and many biological issues remain unresolved.

The topological model developed by Gibson *et al* examined the effect of cell division on cell topology [16]. Patel *et al* further studied the effect of division plane orientation [25]. However, there are no considerations of mechanical and biological properties of cells. Factors such as cell tension and cell proliferation rate are neglected in these studies. Although it was hypothesized that difference in cell proliferation rate can lead to localized topological changes [16], topological models employed in these studies cannot address this issue, as they cannot model differential proliferation. Mechanical models have been used to study the effects of cell growth and cell rearrangement [8], [15]. However, they have not yet been used to study the effect of division plane. The spring-based mechanical model proposed in [11] is limited to mostly plant epidermis, whose cells have simple shapes and stiff walls [7]. This model does not consider cell rearrangements, and therefore cannot be used to study deformable epithelial cells in animals.

At present, the underlying mechanisms that control the variant topological distributions of cells among different species are not well understood. Furthermore, the mechanisms of regulating localized topological changes, for example, at the interface between proliferating and quiescent epithelial cells have not been studied either using topological models or mechanical models. In addition, although mechanical forces are known to accumulate in response to differential proliferation [28]–[30], how such mechanical changes in turn affect cell topology is not known.

Here we study the general mechanisms of regulating cell topology in animal epithelial cells. We use a model that incorporates geometric properties such as cell shape and cell size, as well as mechanical properties such as cell surface tension and cell pressure. In addition, we model the dynamic processes of cell growth and cell rearrangement explicitly. We also study the effects of different orientations of division plane, and the influences of differential proliferation at the interface between proliferating and quiescent epithelial cells. The roles of mechanical forces originating from differential proliferation are also incorporated.

We show that with the consideration of cell growth and cell rearrangement, our model can reproduce commonly observed topological distributions in natural proliferating epithelia in animals, regardless of the orientations of division plane. In addition, we are able to generate different frequencies of hexagonal cells with different orientation schemes of division plane, in agreement with experimental observations and to some degree with previous models. In proliferating epithelial cells interfacing quiescent cells, our results show that localized differential proliferation alone is insufficient to produce the distorted topological changes observed on the boundary between proliferating cells and quiescent cells. However, increased tension, in conjunction with differential proliferation, can reproduce the observed topological changes. We conclude that both division plane orientation and mechanical forces play important roles in the regulation of cell topology during epithelial proliferation. Moreover, cell memory has a significant impact on generating specific topological distributions.

## Results

### Cell Division and Cell Rearrangement Are Sufficient to Generate Commonly Observed Topological Distributions of Cells

We first studied the mechanisms of regulating cell topology in natural proliferating epithelia in animals. Our simulations start from a single cell. For each time step, we increase cell volumes for all cells by a small amount, and divide cells whose volumes exceed a threshold. Therefore, division occurs after a finite number of time steps. During the proliferation phase, three schemes of division plane orientation were introduced: (1) division plane is randomly chosen from a uniform distribution of angles (random scheme); (2) division plane goes through the largest side of a cell (largest side scheme); and (3) rotating the division plane by 90 degrees in successive generations of cell division (orthogonal scheme). More details can be found in Methods. Tissue grows for about 12 rounds of cell divisions from a single cell to >4,000 cells.

**Figure 2:**
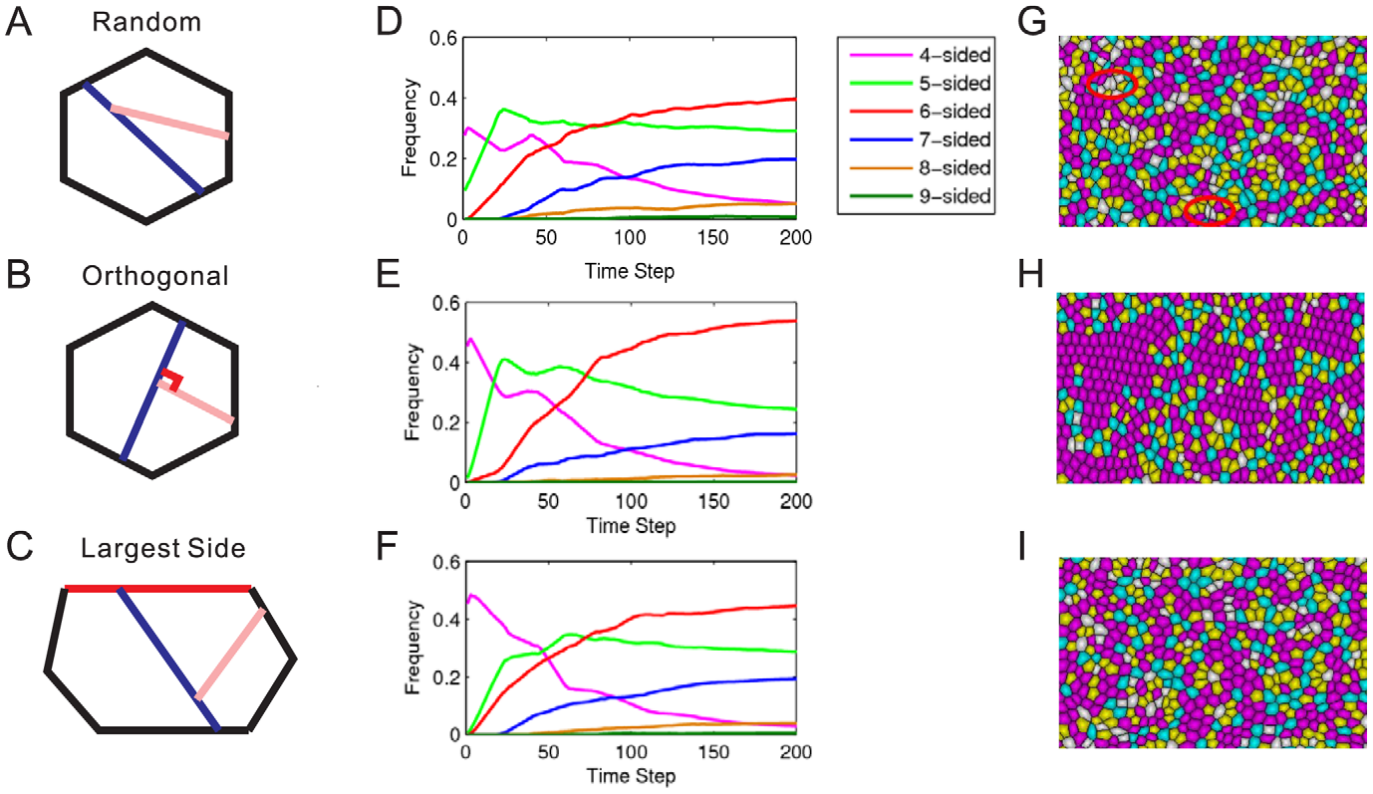
Simulation of different schemes of division plane orientation in natural proliferating epithelia. (A–C) Schematic illustration of division plane orientation. Division plane of the first and the second generation are colored as dark blue and pink, respectively. (D–F) Frequency of cell polygonal types during cell proliferation by three choices of division plane orientation. Random orientation reaches a hexagonal frequency of about 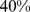. Orthogonal orientation produces a higher percentage of hexagons of about 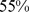. Largest side orientation generates a distribution with a hexagonal frequency of about 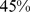. (G–I) Visualization of cell shapes by three division plane orientations. Random orientation generates some cells with irregular shape (marked by red ellipse). Orthogonal orientation produces regular cell shapes with a high percentage of hexagons. Largest side orientation generates regular cell shapes. Pentagons, hexagons, and heptagons are colored yellow, magenta, and blue, respectively. Gray indicates other polygonal types.

Previous experimental and computational studies showed that distributions of cell polygonal types converged to an equilibrium state [8], [11], [15], [16], [25]. In our model, all three schemes of division plane orientation lead to topological distributions with similar features. We can generate the equilibrium distributions of natural proliferating epithelia after 12 rounds of division (Fig. 2D 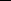
2F). These distributions shared several common features: dominance of hexagonal cells, skewed distributions with more pentagons than heptagons, a mean value of 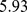 sides with sidedness ranging from 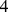 to 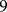 (10-sided cells are extremely rare). Our mechanical model, with cell division and cell rearrangement only, can produce common topological distributions observed in natural proliferating epithelia in animals.

### Regular Cell Shape from the Largest Side and Orthogonal Scheme

We found different orientation schemes of division plane resulted in substantial difference in topological distributions and in regularity of cell shapes. Simulation results from the largest side and random orientation schemes produced similar distributions of cell polygonal types, with about 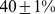 hexagonal cells for random orientation and 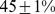 for the largest side orientation. In contrast, orthogonal orientation produced a significantly different distribution, with a frequency of hexagons as high as 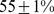 (Fig. 2D 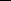
2F). These results show that different schemes of division plane orientation produce quantitatively different distributions of cell polygonal types.

The number of rearrangements in the orthogonal (average = 156) and the largest side (average = 161) were observed to be much lower than in the random (average = 437) scheme (Data shown in Supporting Information S1). In addition, the number of rearrangements increases when the surface tension coefficient 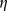 becomes smaller (Data shown in Supporting Information S1). The number of rearrangements in our model refers to the local topological changes and not the large scale cell migration observed in animal tissues.

From visual inspection, we found that both the largest side and orthogonal orientation schemes yielded regular cell shape for individual cells. Overall cell shape for the whole tissue was more regular for the orthogonal scheme, which is consistent with the finding of a higher percentage of hexagonal cells (Fig. 2H). The overall cell shapes generated by random scheme was similar to that from the largest side scheme. However, irregularly shaped cells with extremely small side lengths were present, which are not found in natural proliferating epithelia (Fig. 2G and 2I).

### Different Orientation Schemes of Division Plane Produce Different Frequencies of Hexagonal Cells

**Figure 3:**
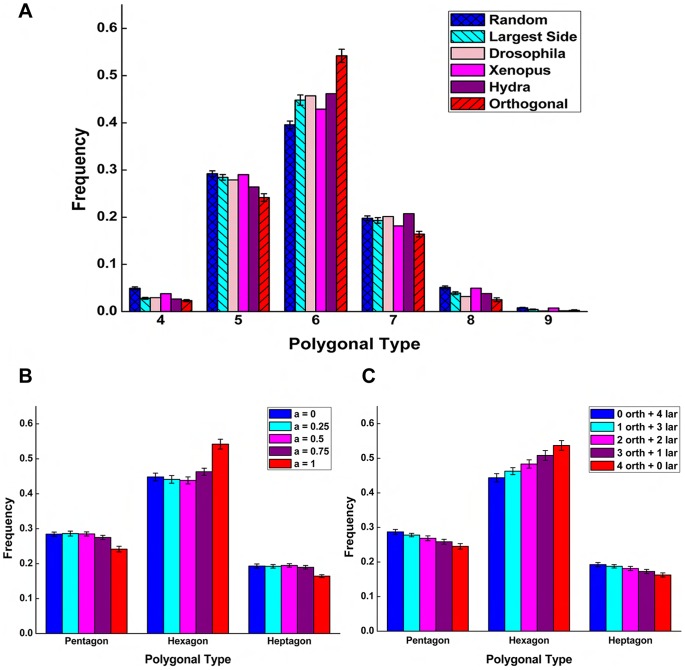
Simulations of topological distributions in proliferating epithelia. (A) The largest side division plane orientation agrees with animal tissues with a peak of around 45% hexagons. Random division plane orientation produces a flatter distribution with a lower percentage of hexagons (40%). Orthogonal division plane orientation generates a distribution with a higher percentage of hexagons (55%). (B) Mitotic cells divide through orthogonal scheme with probability 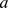, 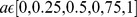. The probability of a cell using orthogonal scheme in division for all its ancestors is 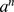, 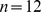. It is close to 0 for 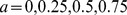, 1 for 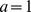. There is no correlation between the topological distribution and different value of 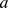. (C) Different combinations of cell types are set initially. Orthogonal cells divide successively using the orthogonal scheme. Topological distribution is linearly correlated to the proportion of initial orthogonal cells.

We compared our simulation results with experimental observations in natural proliferating epithelia in animals. Data from *Drosophila*, *Xenopus*, and *Hydra* gathered by Patel *et al.* can be found in [25]. These species have strikingly similar topological distributions of cell polygonal types, with a peak of approximately 45% hexagons. We found that the largest side division plane orientation matched topological distributions of cells in *Drosophila*, *Xenopus*, and *Hydra*, with the hexagonal frequency of around 45% (Fig. 3A). Orthogonal division plane orientation, on the other hand, generated a higher percentage of hexagons of around 55%. This suggests that different schemes of division plane orientation regulated by local cell properties can reproduce quantitatively variant distributions. This is consistent with previous experimental observations, which demonstrated that distinctly different division rules occur in animals [31], [32].

### Cell “Memory” Is Important for Affecting Cell Topology

In natural epithelia, it is possible that a mixture of different division schemes may lead to distinct topological distributions. To address this issue, we combined orthogonal and the largest side schemes using two different strategies.

In the first strategy, we started simulations from a single cell. Division of mitotic cells are modeled using a mixture of orthogonal and the largest side schemes assigned with different probabilities 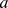 and 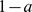, respectively. After around 12 generations of divisions (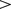
4,000 cells), we found that there was no significant difference in the topological distributions for different probability values (Fig. 3B). The probability of a cell using orthogonal scheme in division for all its ancestors is 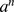 (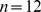) at the end of simulation. It is close to 0 for 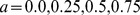, but 1 for 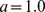. This shows that topological distribution is not altered unless a cell divides successively using the orthogonal scheme. Thus, only when cells divide with orthogonal scheme alone, we obtained the higher percentage of hexagons of about 55%.

In the second strategy, we started simulations from 4 cells with different combinations of cell types (e.g. 1 orthogonal cell and 3 largest side cells). Daughter cells inherit the same cell type and division scheme as their mother cell. After around 10 generations of division (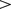
4,000 cells), the probability of an orthogonal cell using orthogonal scheme in division for all its ancestors is 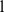. Therefore, the frequency of hexagons is linearly correlated to the proportion of initial orthogonal cells (Fig. 3C).

These different results are related to different properties of these two division schemes. Orthogonal scheme is deterministic, as the orientation of division plane of daughter cells are determined by that of their mother cell. Orthogonal cells receive parental cues, or cell “memory”, for selecting division plane from their ancestors. The largest side scheme only depends on the cell side length at the current time, and which is not directly related to cell “memory”.

### Differential Proliferation Has Little Effects on Topology of Interfacial Proliferating Cells

We then studied proliferating epithelial cells interfacing quiescent cells and investigated the effects of differential proliferation. The inner part of the tissue in our model consists of proliferating cells with non-zero growth rate. These proliferating cells are surrounded by quiescent cells with zero growth rate. Cells at the interface of proliferating cells and quiescent cells are named interfacial proliferating cells (IP cells) and interfacial quiescent cells (IQ cells), respectively. We set the tension coefficient 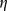 on all boundaries as 1.0.

**Figure 4:**
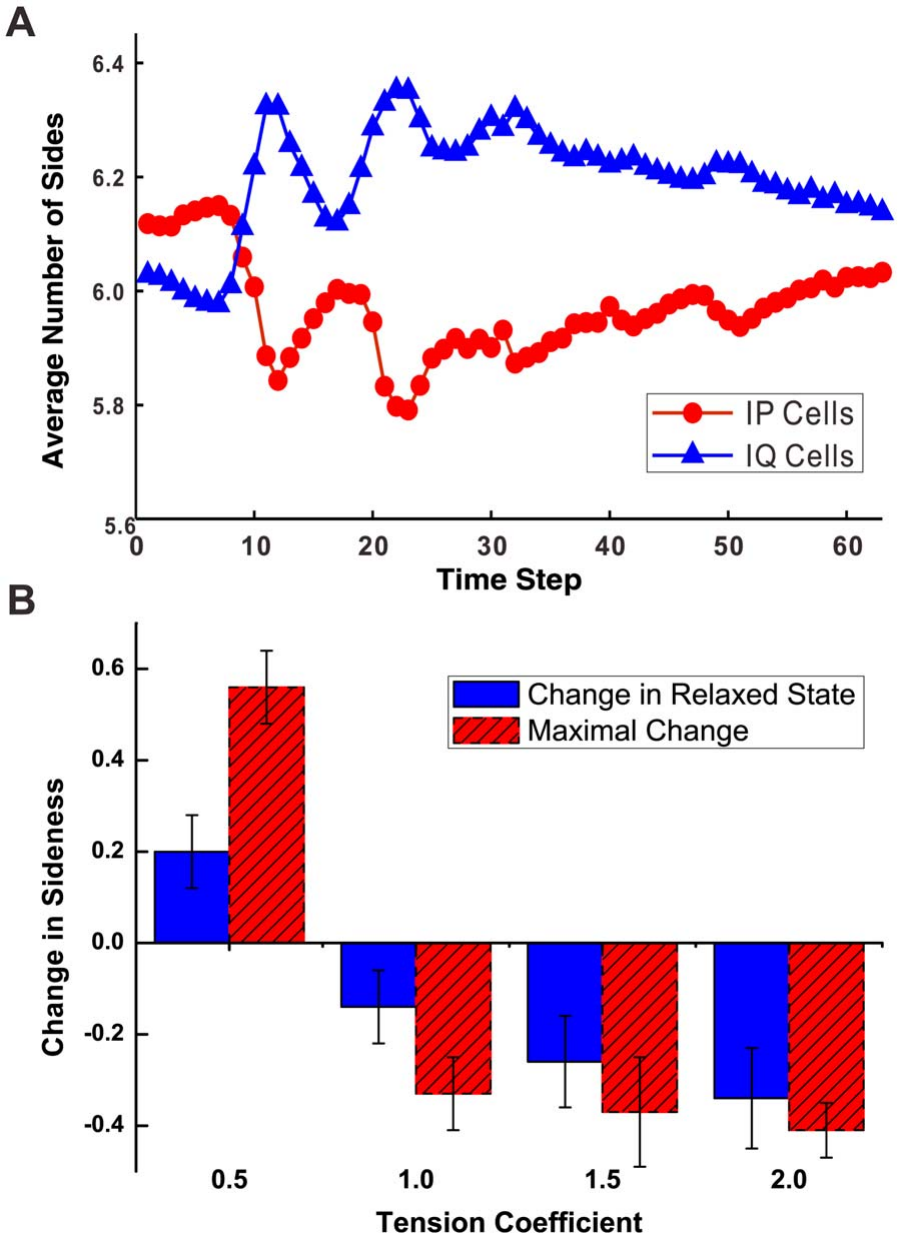
Effect of differential proliferation and mechanical forces. (A) With differential proliferation alone, the average number of sides of IP cells decreases to a limited extent (red), and the average number of sides of IQ cells increases (blue). (B) Change in the average number of sides with different tension coefficients. Increased tension coefficients (1.5, 2.0) on the boundary lead to further reduced average number of sides of IP cells. Decreased tension coefficient (0.5) results in increased average number of sides of IP cells.

At the start of the simulation, the average number of sides of IP cells decreased, with a change in sidedness as much as 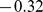. The average number of sides of IQ cells, on the other hand, increased, with a maximum change of 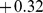 (Fig. 4A). Although there was significant reduction in sidedness at the beginning of the simulation, this effect subsided after proliferating cells underwent additional rounds of division (Approximate 17 time steps are counted as 1 round of division). The reduction in the average number of sides decreased from 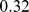 to about 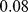 after 3 or 4 rounds of cell division (Fig. 4A).

Overall, these results demonstrate that in the long run differential proliferation alone does not have a significant impact on the topology of IP cells.

### Increased Boundary Tension Produces Fewer Sides in Interfacial Proliferating Cells

Surface tension on the boundary between proliferating and quiescent cells may increase through changes in cytoskeletal microfilaments, intermediate filaments, and cell membrane [33] due to differential proliferation. We modeled this effect by changing the tension coefficient on the boundary between proliferating and quiescent cells. Increased tension coefficient represents more compressional force on the edge. We set the tension coefficient 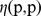 for edges between proliferating cells and 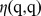 for edges between quiescent cells as 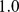. The tension coefficients of edges between proliferating cells and quiescent cells 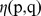 are set to four different values, 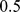, 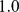, 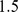, and 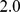, respectively.

We found that when 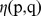 was greater than 1.0, namely, when surface tension increased, the average number of sides of IP cells decreased significantly. The maximal change in the average number of sides at 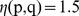, 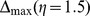, was 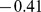. It further decreased when surface tension increased, with 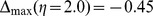 (Fig. 4B). At higher 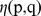, decreases in sidedness fluctuated much less at different generations. After 3 or 4 rounds of cell division, tissue went into a steady state, and the change in average number of sides of IP cells was 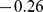 for 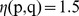. For 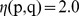, this change was 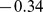. The magnitude of this change in the steady state is larger than 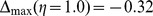 with lower tension coefficient.

In contrast, when surface tension decreased, even though inner cells were proliferating as before, the average number of sides of IP cells increased, with 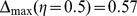 (Fig. 4B). This result was opposite to the decreased average number of sides of IP cells observed in experiments [16]. Our results suggests that the overall decrease in the average number of sides of IP cells is strongly influenced by mechanical forces exerted by cytoskeletal microfilaments, intermediate filaments, and cell membrane [33]. Changes in mechanical forces in opposite directions can lead to differently distorted topological distributions.

We compared simulation results with experimental data for proliferating epithelial cells bounded by quiescent cells [16]. Results obtained with increased boundary tension were consistent with experimental data, in which the change in average number of sides of IP cells was about −0.52. The difference between our simulation results (−0.45) and experiments is likely due to difference in sample variation, as the number of cells used in the experimental study was small (295 cells in 24 clones) and simulation results are obtained from a starting tissue of about 4,000 cells.

The overall consistency between experimental data and simulation results suggests that differential proliferation between proliferating cells and quiescent cells results in changes in mechanical forces. Increased boundary tension contributes significantly in distorting topological distributions of IP cells.

## Discussion

### Effect of Division Plane Orientation

Our simulation results show that different schemes of division plane orientation can account for observed differences in topological distributions in natural proliferating epithelia. An orthogonal orientation scheme can generate a distribution with hexagonal frequency as high as 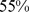. This higher percentage was not seen in previous mechanical models with random orientation scheme, even though a range of parameter choices of mechanical properties was employed [8], [15].

In addition, we found that the probabilistic model does not affect the cell topology significantly, suggesting that the orthogonal orientation scheme needs parental cues, or cell “memory”, in successive generations to affect the overall topological distribution of the tissue. This is because after many divisions during the tissue growth, the probability that each generation of daughter cells used the same cell division scheme as the first ancestor approaches zero for the probabilistic model. We observe that for the deterministic model, the topology of the tissue is directly correlated with the percentage of cells that successively use orthogonal division scheme in each generation. Previous studies have already suggested that biological systems can achieve memory through transcriptional molecules that regulate gene expression [34] or through the maintenance of epigenetic state by stochastic network, which is important for cell differentiation and tissue formation [35]. Through affinity of binding, cooperativity, or multimerization of transcription factors at their binding sites, desired levels of gene expression are maintained overtime in the absence of sharp stimulus, ensuring a long lasting cell memory. Post translational modifications can also produce cues that are maintained in subsequent cell generations [36], although explicit modifications that directly affect division plane are yet to be discovered.

### Effect of Mechanical Forces

Our simulation results also suggest that mechanical forces play important roles in regulating cell topology of animal proliferating epithelia. First, adopting a particular division scheme may reduce the stress exerted on cell edges. This is supported by our observation that the number of rearrangements required in both the largest side (average = 161) and the orthogonal (average = 156) schemes are much smaller than the random (average = 437) scheme (Data shown in Supporting Information S1). Both the largest side and the orthogonal schemes also lead to more regular cell shapes and tissue structure than the random scheme.

Second, increased tension on the boundary between proliferating cells and quiescent cells induced by differential proliferation can significantly affect local cell topology. Our results show that local changes in cell topology can only be achieved when the boundary tension is increased in conjunction with the differential proliferation. Moreover, our results suggest that differential proliferation leads to accumulation in tension on the boundary, and this increased tension plays the most prominent role in distorting cell topology.

In summary, our results suggest that regulation of mechanical forces helps to ensure a regular tissue structure in animal proliferating epithelia. In addition, mechanical forces respond to local changes and control tissue morphogenesis.

### Comparison with Experimental Studies

Our simulation results show that the largest side division plane can generate topological distributions of cells observed in animal proliferating epithelia. We have examined available literatures to assess the relevance of these division schemes in different species and whether other distinct division schemes exist. In *Drosophila* wing, mitotic cells tend to cut the longest axis passing through the neighbor with the least number of sides [26]. This longest axis is correlated with the smallest neighbor/largest side scheme [25]. Our simulations produce similar topological distributions as reported in [25], [26]. In *Xenopus* egg and adult *Hydra*, it was found experimentally that cell shapes guide spindle orientations towards the long axis [37], [38].

### Further Applications of Our Mechanical Model

Our mechanical model provides a platform to study other problems in tissue morphogenesis, for example, the effect of oriented divisions, molecular gradients, and mechanical forces. We have concentrated our study on proliferating epithelia that have isotropic growth. It would be interesting to study proliferating epithelia with anisotropic growth. There is considerable evidence that oriented cell division is relevant for tissue morphogenesis in a variety of organisms [2], [3], [39]. The oriented tension is also found during tissue elongation in *Drosophila* [40]. These oriented division planes and mechanical forces may have important roles during organ development. Molecular gradients are also important for cell growth and cell division. Concentration of growth factor receptors (such as EGFR) and nutrients (such as oxygen) can significantly affect cell proliferation [41], [42]. Secreted morphogens (such as Dpp) are known to play important roles in controlling organ size and pattern formation in *Drosophila* [43], [44]. All these factors can be incorporated in our model. For example, we can introduce a morphogen-based growth rate (increasing volume) in our cells with different mechanistic models on how the spatial distribution of the morphogen controls the growth of individual cells. We can also model spatial distribution of morphogen gradients using discretized finite difference equations. Furthermore, it will be interesting to study these chemical signals in conjunction with mechanical forces. Regulation of cell growth and division also has a fundamental role in cancer progression [4], [5]. Both experimental and theoretical studies showed that cell-cell and cell-matrix interactions had significant effects on cancer invasion and migration [9], [45]–[48]. It is likely that investigation of the effects of these mechanical forces will be useful for understanding cancer progression.

### Conclusions

We have used a two-dimensional mechanical model to study the mechanisms of regulating cell topology both in natural proliferating epithelia in animals and proliferating epithelial cells interfacing quiescent cells. This model is able to take into account geometric properties of single cells, such as cell shape and size, as well as mechanical properties such as tension and pressure. By comparing experimental data and simulation results, we find that, regardless of division plane orientations, our model can produce the commonly observed topological distributions in natural proliferating epithelia. In addition, with different schemes of division plane orientation, our model generates quantitatively different distributions of cell polygonal types. In proliferating epithelial cells bounded by quiescent cells, our results show that, only increased boundary tension with differential proliferation can significantly decrease the average number of sides of interfacial proliferating cells. We conclude that both division plane orientation and mechanical forces play important roles in regulating cell topology during epithelial proliferation. Moreover, cell memory may significantly affect the overall topological distribution.

## Methods

### Geometric Model of Cells

**Figure 5:**
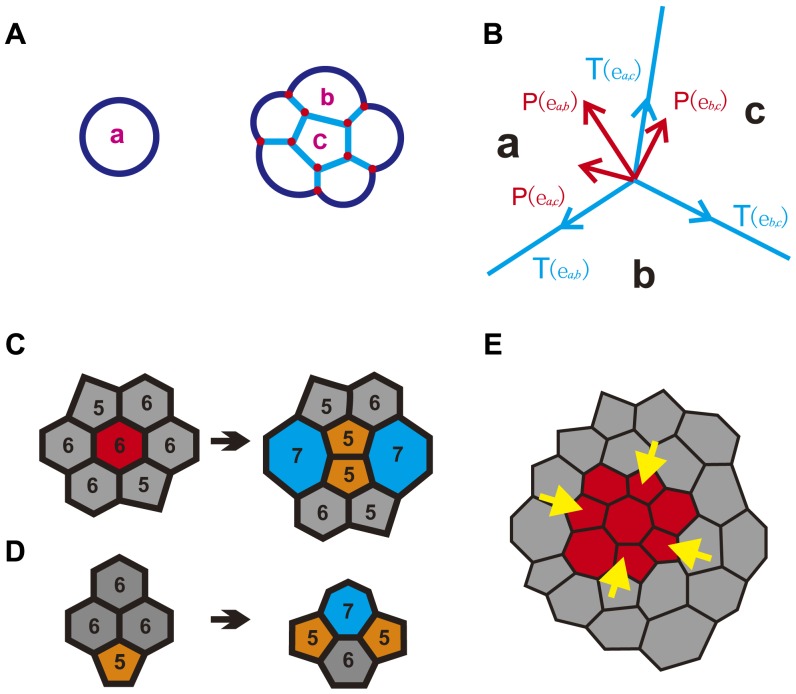
Cellular model for simulations of cell topology. (A) Geometric representation of cells. Cell *a*, in isolation, is modeled as a disk. Cell *b*, at the boundary of a tissue, is modeled as a disk segment. Cell *c*, completely surrounded by other cells, is modeled as a polygon. Inner edge (light blue) is a straight line segment, and outer edge (dark blue) is an arc or a circle. Vertex (red dot) is the junction point of three edges. (B) Mechanical forces acting at the junction vertex of three cells *a*, *b*, and *c*. Tension is tangential to the edge (blue). Pressure is normal to the edge (red), from the cell with higher pressure to the cell with lower pressure. (C) Cell topology is affected by cell division. After division of the mitotic cell (red, hexagon), daughter cells (orange, pentagon) lose sides on average (5
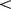
6). Two neighboring cells gain one side each (blue, heptagon), leading a transition from hexagon to heptagon. (D) Cell topology is affected by cell rearrangement. Three hexagons (gray) and one pentagon (orange) transfer to one hexagon (gray), one heptagon (blue), and two pentagons (orange). The distribution of cell polygonal types changes. (E) Epithelial tissue with differential proliferation. Internal proliferating cells (red) grow outward, pushing the outside quiescent cells (gray). Outside quiescent cells tend to stay at their original positions, compressing the inner proliferating cells (yellow arrows).

Epithelial monolayer is represented by a two-dimensional sheet composed of tightly adherent neighboring cells. Individual cells within an epithelia are approximately polygonal in shape [12]. We use a previously developed mechanical model (detailed in Supporting Information S1) to study the mechanisms of regulating cell topology in proliferating epithelia [49], [50]. This model captures the geometric properties of cells, including area, length, internal angles, as well as the topological connectivity between cells. Briefly, a biological cell is presented by the combination of three types of geometric elements (Fig. 5A):

#### Cell

Cell is a spatial region representing the volume of a cell. It is a disk when in isolation, but a disk segment when at the boundary of a tissue. It is represented as a polygon when it is completely surrounded by other cells. Cells can have different sizes.

#### Edge

Edge is the boundary of a cell. There are two types of edge: *inner edge* and *outer edge*. An *inner edge* is modeled as a straight line segment when two cells are connected. An *outer edge* is modeled as an arc or a circle when it represents the cell boundary between a cell and the outside medium.

#### Vertex

Vertex is the junction point of three edges. In our model, we assume no more than three cells can interact. That is, no more than three edges can meet at a vertex.

### Mechanical Forces

Cell movement and subsequent rearrangement in an epithelial sheet are determined by mechanical forces generated in a cell [13], [51]. These mechanical forces are distributed throughout the cytoskeleton system and enable a cell to adhere to neighboring cells and the extracellular matrix [52], [53]. These mechanical forces are modeled as tension and pressure forces in our model.

#### Tension

Tension represents compression forces acting on a cell. It originates from cytoskeletal microfilaments, intermediate filaments, and cell membrane [33]. For the inner edge 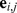 between cells 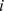 and 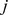, the tension force is always tangential to the edge 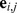 (Fig. 5B):
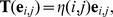
where 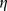 is the tension coefficient, which may depend on the cell types of both cells, and 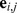 is the unit vector in the direction of shortening edge 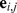.

#### Pressure

Pressure represents the expansion forces. It arises mainly from microtubules and extracellular matrix [33]. For the inner edge 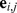 between cells 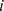 and 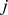, the net pressure force is proportional to the difference in pressure in cell *i* and *j*. It is in the direction normal to the edge 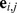, from the cell with higher pressure to the cell with lower pressure (Fig. 5B).

In our model, forces act on vertices. The net force on each vertex 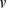 can be decomposed as
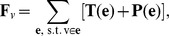
which sums over all the edges 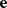 ending in vertex 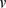. Here 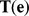 and 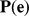 are the forces acting on edge 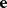 through cell wall tension and intracellular pressure, respectively.

### Topological Changes during Cell Proliferation

Mechanical forces can directly change the geometry of individual cells, which often lead to topological changes in overall cellular and tissue pattern. This is reflected by changes in the distribution of cell polygonal types. We incorporate two biological processes that affect the topological changes during cell proliferation in our model: *cell division* and *cell rearrangement*.

#### Cell Division

When a cell divides, a new edge is added in the mother cell, with two new vertices added at the end points of the new edge (Fig. 5C). During division, the division plane passes through the mid point of the selected edge and the mass center of the cell. Degeneracy of a new vertex coinciding with an existing vertex is handled by insisting that a new infinitely small edge is added, along with the new vertex. Both the number of contacts and the connectivity of cells change during cell division. Multiple generations of cell division can significantly alter the cell topology in the tissue.

#### Cell Rearrangement

When two originally disconnected cells expand faster than their neighbors, they will come into contact and push away the two previously connected neighboring cells (Fig. 5D). In this case, we remove the edge between two previously connecting cells, and add a new edge between two cells that now come into contact. The total number of cell sides for the whole tissue remains the same, but the connectivity of cells changes during cell rearrangement.

### Orientation of Division Plane

We also study the effects of the orientation of division plane during cell division. In our model, cell division occurs when the size of a cell is doubled. A new cell wall, which is described as *division plane*, is created. It passes through the mass center of the mother cell, and each daughter cell inherits approximately half of the volume of the mother cell. Both experimental and theoretical studies show that cells experience symmetric divisions to preserve tissue structure in simple proliferating epithelia [11], [25], [54]–[56].

We hypothesize that the orientation of division plane contributes to the variant topological distributions in natural proliferating epithelia among different species. Physically, the cortical tension along the edge in mitotic cells can influence division plane orientation [32], [57]. Division plane orientations in mother cell and the daughter cells may also be correlated [31], [58]. To study this effect, we examine three schemes of division plane orientation: *random*, *largest side*, and *orthogonal* scheme (Fig. 2A 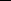
2C).

#### Random Scheme

Division plane is randomly chosen from a uniform distribution of directions in all angles. This scheme models the scenario that the orientation of division plane plays no significant role in cell topology. It also serves as a control model.

#### Largest Side Scheme

Division plane cuts through the largest side of a cell. This is based on the observation that the orientation of mitotic spindle in human cells lies almost parallel to the largest side, and the largest side of the cell is often split during cell division in order to reduce the stress on the edge [32].

#### Orthogonal Scheme

Division plane is rotated by 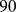 degrees in the successive generations. This strategy is commonly seen in plants [31], [58].

### Mechanical Forces in Proliferating Cells Interfacing Quiescent Cells

In the experimental study by Gibson *et al.*, clones of rapidly proliferating cells are surrounded by quiescent cells. The distribution of cell polygonal types for proliferating cells interfacing quiescent cells was found to be significantly different from that in the natural proliferating epithelia [16].

We hypothesize that differential proliferation can lead to changes in mechanical forces acting on cells, which in turn significantly affect cell topology. Rapidly proliferating cells in the interior of tissue push the outside quiescent cells, which at the same time, the outside quiescent cells tend to stick to their original positions. Consequently, the overall behavior is as if the inner proliferating cells experienced compression forces (Fig. 5E), and the surface tension on the boundary between these proliferating cells and quiescent cells increases. We study the validity of this hypothesis.

### Simulation Methodology

We study the cell topology for both natural proliferating epithelia and proliferating epithelial cells interfacing quiescent cells.

#### Natural Proliferating Epithelia

We simulate the proliferating process of natural proliferating epithelia using the following procedure. We found that in a homogeneous tissue, differences in mechanical properties do not affect their topological distributions (Data shown in Supporting Information S1). This is consistent with results from a previous study [11]. We start simulations from a single cell. We increase cell volumes (random amount between 1%–8%) during each time step so that all cells in the tissue grow at the same time. Mitotic cells are selected as cells whose volume exceed a threshold value, and are divided into two daughter cells, with approximately equal volume. Different schemes of division plane orientation (random, largest side, and orthogonal scheme) are applied in the simulation during the whole proliferating process. Tissue grows for around 12 generations of cell divisions (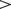
4,000 cells). For each scheme of division, simulations are repeated for 10 times. We record the topological distributions through the time, and take the averages as our results.

#### Proliferating Epithelial Cells Interfacing Quiescent Cells

Here we start simulations with the tissue in equilibrium state, containing about 4,000 cells (data from simulated natural proliferating epithelia). Inner part of the tissue are assigned as proliferating cells with non-zero growth rate, whereas outer part are assigned as quiescent cells with zero growth rate (based on experimental studies of [16]). The growing process is the same as the procedure in natural proliferating epithelia. The largest side division plane orientation is selected as it can produce the topological distribution in *Drosophila* [49]. Different tension coefficients 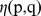 on the boundary are employed to study the effect of mechanical forces (0.5, 1.0, 1.5, 2.0, with 1.0 being default). 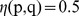 indicates decreased compressional force, while 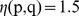 and 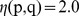 represent increased compressional forces. We examine the topological distributions of cells at the interface of proliferating cells and quiescent cells during the proliferating process. For each choice of tension coefficient, we run simulations for 5 times and take the average as our results.

Our model is implemented with C++. Simulations were performed with 64-bit Linux cluster. Software is available upon request.

## Supporting Information

Supporting Information S1 Supplementary Information.
(PDF)
