# Supplementary material for: Mechanisms of Regulating Cell Topology in Proliferating Epithelia: Impact of Division Plane, Mechanical Forces, and Cell Memory
Source: PLoS One. 2012 Aug 17;7(8):e43108. doi: 10.1371/journal.pone.0043108 (PMC3422310; doi:10.1371/journal.pone.0043108)
Supplement: Supporting Information S1 — Supplementary Information. (PDF) [file pone.0043108.s001.pdf]

Supplementary Information

	Frequency of Polygonal Type		
Tension Coefficient	Pentagon	Hexagon	Heptagon
1.0	0.24	0.55	0.16
1.5	0.24	0.54	0.16
2.0	0.24	0.54	0.16

Table S 1: Frequency of polygonal type in the equilibrium state with different tension coefficients. The orthogonal scheme is employed in simulations.

	Random		Largest Side		Orthogonal	
Trial No.	Rearrangements	Division	Rearrangements	Division	Rearrangements	Division
1	423	4162	150	4171	148	4189
2	435	4123	160	4180	149	4152
3	454	4184	173	4177	138	4135
4	430	4109	148	4178	176	4166
5	451	4135	159	4159	143	4123
6	412	4144	179	4197	147	4186
7	433	4132	156	4119	165	4180
8	442	4151	185	4179	166	4155
9	444	4148	152	4148	158	4186
10	448	4172	141	4185	149	4122
Average	437	4146	161	4169	156	4159

Table S 2: The number of divisions and rearrangements observed using random, largest side, and Orthogonal division schemes, with η=1.

η	0.5		1.0		1.5		2.0	
Trial No.	Rearrangements	Divisions	Rearrangements	Divisions	Rearrangements	Divisions	Rearrangements	Divisions
1	224	4170	148	4159	130	4197	113	4180
2	234	4170	138	4189	125	4151	121	4133
3	255	4139	173	4135	97	4161	111	4159
4	254	4141	176	4166	100	4189	95	4116
5	228	4125	143	4123	149	4146	111	4162
6	232	4194	147	4186	121	4128	95	4161
7	238	4114	165	4180	108	4172	95	4169
8	222	4161	166	4155	115	4149	118	4155
9	245	4192	158	4186	133	4159	111	4105
10	237	4154	149	4122	129	4152	105	4144
Average	237	4156	156	4159	121	4160	108	4148

Table S 3: The number of divisions and rearrangements observed with different values of η using the orthogonal division scheme.

Geometric Model of Cell

The underlying physics of our two-dimensional cellular model is that of a well-studied topic of surface energy minimization, also called *bubble formation* (1; 2; 3). In this model, each cell minimizes its surface energy, as prominently observed in the developing retina of *drosophila*, where differential expression of N-cadherin leads to the formation of an overall shape that minimizes their surface contact with surrounding cells (4).

Formally, a biological cell is represented by the combination of three types of geometric elements (Fig S1). First, a *geometric cell* $\boldsymbol{c}_i$ is a spatial region representing the volume of cell i (Fig S1a). It is a disk when in isolation, but can be a disk segment when the cell is contacting other cell(s). It can take the shape of the union of a polygon and one or more disk segment(s), when there are multiple contacting cells in the surroundings, and at the same time there is one or more free boundaries (Fig S1b-c). When completely surrounded by other cells, it takes the form of a polygon (Fig S1d). Cells can have different sizes.

Second, *edges* represent the boundaries of a cell. There are two types of edges: *outer edges* $\boldsymbol{e}_i$ for cell i and *inner edges* $\boldsymbol{e}_{i,j}$ between cell i and cell j (Fig S1b). An *outer edge* $\boldsymbol{e}_i$ is an arc or a circle, and represents the boundary between cell $\boldsymbol{c}_i$ and the outside medium (denoted as $\boldsymbol{c}_0$). $\boldsymbol{e}_i$ is a full circle when the cell exists in isolation, but becomes one or more arcs if the medium does not fully surround the cell. An *inner edge* $\boldsymbol{e}_{i,j}$ occurs when a cell $\boldsymbol{c}_i$ is in contact with another cell $\boldsymbol{c}_j$. Their shared boundary is a face with constant surface curvature (1), but is modeled as a straight line segment here. An inner edge appears twice, once for each of the neighboring cell, with the order of the two indices reversed. This reflects the fact that each cell has a separate wall.

Third, *vertex* is the junction point of three edges. When two cells $\boldsymbol{c}_i$ and $\boldsymbol{c}_j$ make contact (Fig S1b), their outer edges (arcs) $\boldsymbol{e}_i$ and $\boldsymbol{e}_j$ intersect at two vertices, $\boldsymbol{v}_{i,0,j}$ (indices in clockwise direction) and $\boldsymbol{v}_{j,0,i}$, which are also the two end-points of the inner edge $\boldsymbol{e}_{i,j}$ (Fig S1b). When three cells $\boldsymbol{c}_i, \boldsymbol{c}_j$ and $\boldsymbol{c}_k$ intersect (Fig S1c), they form a single vertex $\boldsymbol{v}_{i,j,k}$. In our two-dimensional model, we assume no more than three cells can intersect as seen in soap

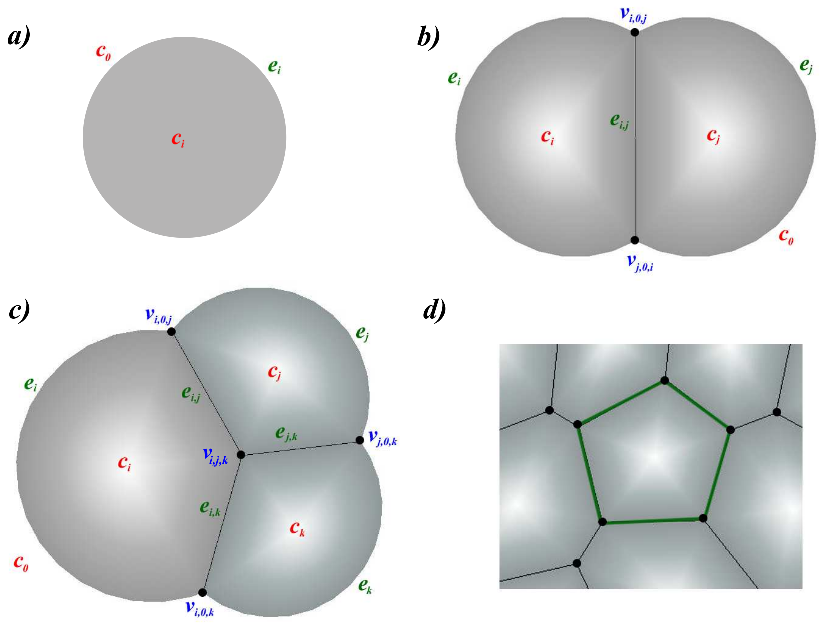

Figure S 1: Two-dimensional cell model. a) An isolated cell is modeled as a disk. b) A cell is modeled as a disk segment when contacting other cell(s). An *outer edge* $\boldsymbol{e}_i$ is an arc or a circle, representing the boundary between cell $\boldsymbol{c}_i$ and the outside medium (denoted as $\boldsymbol{c}_0$). An *inner edge* $\boldsymbol{e}_{i,j}$ occurs when a cell $\boldsymbol{c}_i$ is in contact with another cell $\boldsymbol{c}_j$. Their shared boundary is modeled as a straight line segment. When two cells $\boldsymbol{c}_i$ and $\boldsymbol{c}_j$ make contact, their outer edges (arcs) $\boldsymbol{e}_i$ and $\boldsymbol{e}_j$ intersect at two vertices $\boldsymbol{v}_{i,0,j}$ and $\boldsymbol{v}_{j,0,i}$, which are also the two end-points of the inner edge $\boldsymbol{e}_{i,j}$. c) When three cells $\boldsymbol{c}_i, \boldsymbol{c}_j$ and $\boldsymbol{c}_k$ intersect, they form a vertex $\boldsymbol{v}_{i,j,k}$. d) A cell completely surrounded by other cells is represented as a polygon.

bubbles (1; 2; 3). That is, no more than three edges can meet at a vertex.

For a tissue consisting of n cells, we denote the set of cell centers as $\boldsymbol{Z} = \{z_1, \cdots, z_n\}$, where $z_i \in \mathbb{R}^2$ is the coordinates of the center of cell i. The set of edges is denoted as $\boldsymbol{E} \equiv \{\boldsymbol{e}_i\} \cup \{\boldsymbol{e}_{i,j}\}$, and the set of vertices is denoted as $\boldsymbol{V} = \{\boldsymbol{v}_{i,j,k}\}$. The overall state $\boldsymbol{S}$ of a tissue with n cells is defined as: $\boldsymbol{S} = (\boldsymbol{Z}, \boldsymbol{E}, \boldsymbol{V})$. It fully determines the geometric pattern of the tissue formed by these n cells.

Physical Model of Cell and Cell Growth

Stationary Model We model cells with the assumption that they are in a stationary state, in which changes are slow, and all forces in the system at every moment are balanced out by each other.

We use discrete time steps to model incremental changes of cell volume, which is dictated by the underlying biology, *e.g.* cell birth, cell death, cell growth and shrinkage, and changes of cell wall properties. In our model, we assume the energy of the cells reaches a minimum at the state $\boldsymbol{S}$:

$$E(\boldsymbol{S}) = E(\boldsymbol{X}, \boldsymbol{E}, \boldsymbol{V}) = \min .$$

Forces exerting on the system of cells at state $\boldsymbol{S}$ have a zero net sum. We model the mechanical forces using only the vertices. The geometry of the whole system, including the edges and cells, and the forces in each cell, all will follow once the vertex set $\boldsymbol{V}$ is specified. We have:

$$\boldsymbol{F}(\boldsymbol{S}) \equiv \boldsymbol{F}(\boldsymbol{V}) = \frac{dE(\boldsymbol{V})}{d\boldsymbol{V}} = 0.$$

Mechanical Forces A number of physical forces exist inside a cell. Cytoskeletal microfilament (9; 10; 11), intermediate filaments (12), and cell membrane all exert *compression forces* on a cell. In addition, there exists adhesion or alternatively repulsion force between cells. These forces are summed up and modeled as a *tension force* that exerts along the direction $\boldsymbol{e}_{i,j}$ of inner edge (interior cell boundary), or along the tangent direction of outer edge $\boldsymbol{e}_i$ (free cell boundary).

There are also *expansion forces* in a cell. These include those from microtubles (13; 10; 14; 11) and the extracellular matrix (ECM) (15). We model these forces as a *pressure force* that acts along the direction normal to the edge. Pressure force only exists for an inner edge and not for arc/outer edge. Tension force exists at vertices due to both inner and outer edges.

In our model, cells minimize their energy and take upon the appearance and behavior of soap bubbles (4). According to the soap bubble model, cell walls take the shape of constant mean curvature (CMC) surfaces under fixed volume and pressure conditions (1). The most notable examples are spherical shape and biconcave erythrocyte shape (16). We therefore model cells as intersecting circular disks.

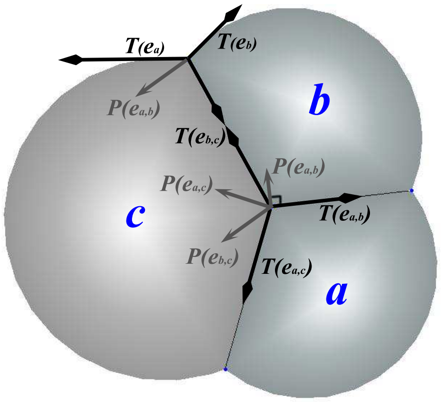

Figure S 2: Forces applied to the junction vertex of three cells a, b, and c. The *tension force* $\boldsymbol{T}(\boldsymbol{e}_{i,j})$ exerts along the direction $\boldsymbol{e}_{i,j}$ of an inner edge (interior cell boundary), or along the tangent direction of outer edge $\boldsymbol{e}_i$ (free cell boundary), where (i, j) are the two indices of cells a, b, or c. The *pressure force* $\boldsymbol{P}(\boldsymbol{e}_{i,j})$ acts along the direction normal to the cell boundary, in the direction from the cell with higher pressure to that with lower pressure.

Physical forces are modeled to act at vertices (Fig S2). For inner edges, although the physical cell wall will adopt a curved surface under the bubble model, the curvature is small and we simplify it as a straight line segment. The physical forces originally tangent to the curved surface are now decomposed into two components for the straight line segment. They are the tension force $\boldsymbol{T}(\boldsymbol{e}_{i,j})$ and the pressure force $\boldsymbol{P}(\boldsymbol{e}_{i,j})$. Tension force acts in the direction

of shortening the edge, and pressure force follows the direction of the difference of pressure in two cells sharing the edge.

For outer edges, we take the curved surface into account and model it as an arc. In this case, the physical force tangent to the curved surface is modeled directly as the tension force $\boldsymbol{T}(\boldsymbol{e}_i)$ for this cell, in the direction of shortening the arc. The pressure inside the boundary cell containing this outer edge determines the curvature of the outer edge, and therefore determines the tangential direction of the tension force.

Formally, the forces applied to a vertex v in $\boldsymbol{V}$ can be decomposed as

$$\mathbf{F}_v = \sum_{\boldsymbol{e}, \text{s.t.} v \in \boldsymbol{e}} [\mathbf{T}(\boldsymbol{e}) + \mathbf{P}(\boldsymbol{e})], \tag{1}$$

which sums over all edges $\boldsymbol{e}$'s ending in the vertex v. Here $\mathbf{T}(\boldsymbol{e})$ and $\mathbf{P}(\boldsymbol{e})$ are the forces acting on edge $\boldsymbol{e}$ through cell wall tension and intracellular pressure, respectively.

For the edge $\boldsymbol{e}_{i,j}$ between cells i and j, the tension force is always tangential to the edge $\boldsymbol{e}_{i,j}$:

$$\boldsymbol{T}(\boldsymbol{e}_{i,j}) = \eta(i,j)\boldsymbol{e}_{i,j},$$

where η is the tension coefficient, which may depend on the cell types of both cells, and $\boldsymbol{e}_{i,j}$ is the edge vector. We assume $\boldsymbol{e}_{i,j}$ is in the direction of shortening $\boldsymbol{e}_{i,j}$, otherwise, we add a coefficient "-1" in front of this formula.

Pressure induced force is in the direction normal to the inner edge:

$$\boldsymbol{P}(\boldsymbol{e}_{i,j}) = (P_i - P_j)|\boldsymbol{e}_{i,j}|\boldsymbol{n}_{(i,j)},$$

where P_i and P_j are the pressures in two cells, $|\boldsymbol{e}_{i,j}|$ is the length of the inner edge, and $\boldsymbol{n}_{(i,j)}$ is the unit vector normal to the edge in the direction from the cell with higher pressure to the cell with lower pressure. Although pressure force exerts on the whole inner edge, we decompose it equivalently to the two end vertices, each distributed with $\frac{1}{2}$ of the total pressure force $\boldsymbol{P}(\boldsymbol{e}_{i,j})$.

For an outer edge $\boldsymbol{e}_i$ of cell i, the tension force acting on a vertex $\boldsymbol{v}$ is always tangential to the arc $\boldsymbol{e}_i$, in the direction $\boldsymbol{t}_v$ of shortening $\boldsymbol{e}_i$:

$$\boldsymbol{T}(\boldsymbol{e}_i) = \eta(i,0)|\boldsymbol{e}_i|\boldsymbol{t}_v.$$

Here 0 denotes the outside medium, $\eta(i,0)$ is the tension coefficient of the outer edge of cell i. The internal pressure is compensated by pressure due to the curvature of the arc of the cell boundary. Therefore, pressure induced force is zero. The value of the pressure inside an outer cell i itself is determined by the curvature of the edge:

$$P_i = \eta(i,0)/r_i.$$

Here r_i is the radius of the arc.

In general, forces at the two vertices of an edge can be in different direction, which can result in displacement of the edge. The movement of an edge is the result of the volume change of the cell. The new position of the edge forms an irregular quadrilateral with the edge before the movement. We distribute this volume change to the two vertices, each with a triangle. This volume change happens in each time integration step (Fig S3).

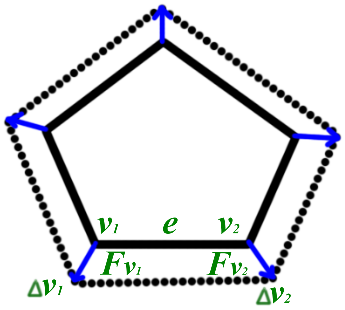

Figure S 3: Diagram representing the forces involved during cell growth. Vertices v_1 and v_2 are moved by Δv_1 and Δv_2 due to the forces $\mathbf{F}_{v_1}$ and $\mathbf{F}_{v_2}$, during the growth process respectively. $\boldsymbol{e}$ is the edge connecting the two vertices. Its length is $|\boldsymbol{e}|$.

Cell Growth We assign different volume changes to individual cells to reflect different stage of cell cycle. For cell i, when $\Delta V_i > 0$, cell i grows. When $\Delta V_i < 0$, cell i shrinks. When $\Delta V_i = 0$, cell i stays in a steady state.

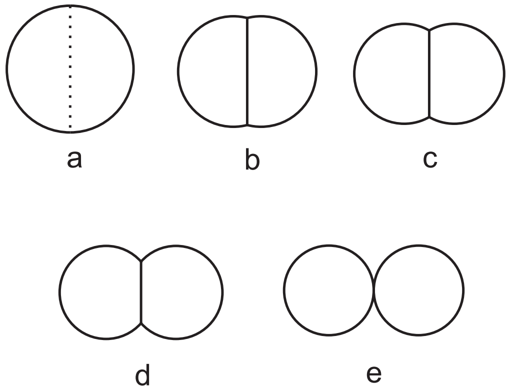

Figure S 4: Cell geometry is determined by the ratio of the tension coefficients. a) when $\eta(i,j) = 0$, there is no tension on an inner edge, and it can be regarded as an imaginary cell wall; b) when $\eta(i,j) = 0.5\eta(i,0)$, there is a strong adhesion force between the two cells; c) when $\eta(i,j) = \eta(i,0) = \eta(j,0)$, the two cells behave as if physically they have the same wall; d) when $\eta(i,j) = 1.5\eta(i,0)$, there is a weak adhesion force between the two cells; e) when $\eta(i,j) \geq 2\eta(i,0)$, The two cells have no adhesion and behave like soccer balls. Adding an inner wall would be more costly, as it is equivalent to adding two outer walls. In this case, the overall energy of the two cells is not reduced.

Cell Shape In our model, the cell walls take the shape of constant mean curvature surfaces under fixed volume and pressure conditions. Physically, each cell has its own wall, and the surface tension $\eta(i,j)$ at an inner edge depends on the properties of both cell walls. The final shape of a cell depends on the ratio of tension coefficient $\eta(i,j)$ for inner edge and $\eta(i,0)$ for the outer edge. When $\eta(i,j) = 0$, there is no tension on an inner edge, and it can be regarded as an imaginary cell wall. When $\eta(i,j) = 0.5\eta(i,0)$, there is a strong adhesion force between the two cells. When $\eta(i,j) = \eta(i,0) = \eta(j,0)$, the two cells behave as if physically they have the same wall. When $\eta(i,j) \geq 2\eta(i,0)$, adding an inner wall would be more costly,

as it is equivalent to adding two outer walls. In this case, the overall energy of the two cells is not reduced. The two cells therefore have no adhesion and behave like soccer balls.

Calculating Forces For cell i that experiences cell volume change ΔV_i at time step t, the net force at each of its vertices can be calculated based on the assumption of stationary state:

$$\Delta V_i = \frac{1}{2}\sigma \sum_{\boldsymbol{e}} \sum_{v(\boldsymbol{e})} |\boldsymbol{F}_{\mathrm{v}(\boldsymbol{e})} \times \boldsymbol{e}|. \tag{2}$$

Here the coefficient of 1/2 represents the change in volume of one of the two triangles formed by the division of the irregular quadrilateral (Fig S3). The summation is over all edges $\boldsymbol{e}$ of cell i and over both end vertices $\{v(\boldsymbol{e})\}$ of each edge, "$\times$" represents vector cross product, and σ is the constant for the integration step. Eqn (2) is solved to obtain the forces at each vertex.

Updating vertex position. At time $t+1$, the location of a vertex $\boldsymbol{v}_i$ after the volume change is updated to:

$$\boldsymbol{v}_i(t+1) = \boldsymbol{v}_i(t) + \sigma \boldsymbol{F}_{\mathrm{v}_i}(t), \tag{3}$$

where $\boldsymbol{v}_i(t)$ and $\boldsymbol{v}_i(t+1)$ are locations of vertex i before and after the time step, respectively. Time t is an integer representing the number of time steps since the initial time, σ is a constant that controls the convergence rate towards stationary state, and $\boldsymbol{F}_{\mathrm{v}_i}$ is the net force exerting at vertex location $\boldsymbol{v}_i$.

Algorithm for Calculating Stationary State We assume all cells exist in stationary state at the end of each time step of simulation. Cells can grow or shrink during a time step. The amount of volume changes are assigned from models of underlying biological process. The altered cell volume leads to movement of cell boundaries. In addition, cell wall properties such as the surface tension coefficients may also be different at different time steps.

All these changes are introduced in increments of small fractions. For each increment, we solve Eqn (2) to obtain the updated forces. We then move the vertices using Eqn (3) to their new locations. After the final increment of volume or cell property change was introduced, we continue iterations with constant volume and constant cell properties. Vertices are further moved until the system relaxes and reaches stationary state, and a balance of the forces is established (Eqn 2). This is the same as applying a gradient search method to find a local minimum of system energy of the cells (17). We then take the geometric patterns of the cells at this state as that of time step $t+1$.

The procedure for computing the stationary state of the cell pattern after one time step is illustrated in Algorithm 1. Here $\boldsymbol{F}_{v_i}$ are the forces acting on vertex i; $\boldsymbol{V}(t) = (\boldsymbol{v}_1(t), \cdots, \boldsymbol{v}_m(t))$ is the vector of coordinates of all of the m vertices at time t; $\Delta\mathcal{V}(t) = (\Delta V_1, \cdots, \Delta V_m)$ is the vector of desired volume changes associated with the vertices for all cells at time t, $\Delta\eta$ is the vector representing desired changes in the cell properties (*e.g.*, cell tension coefficients, cell color) for all cells at time step t. The output is the new coordinates of the vertices $\boldsymbol{V}(t+1)$ at time step $t+1$.

Algorithm 1 UpdateCellPattern ($\boldsymbol{V}(t)$, $\Delta\mathcal{V}(t)$, $\Delta\eta(t)$, σ, k)

$//\epsilon$: Threshold of forces
$//k$: Parameter for step size in incremental volume change.
while $\boldsymbol{F}_v > \epsilon$ for any vertices or $\Delta\mathcal{V}(t)$ not reached yet **do**
  Solve Eqn 2 to obtain updated forces $\boldsymbol{F}_v$ for all vertices.
  if desired amount of changes in $\Delta\mathcal{V}(t)$ not reached yet **then**
    Introduce incremental changes $\Delta\mathcal{V}(t)/k$,
  end if
  Obtain new positions for all vertices using $\boldsymbol{v}_i' = \boldsymbol{v}_i + \sigma\boldsymbol{F}_{v_i}$
  Update topological changes if required
end while
Assign $\boldsymbol{v}_i(t+1) = \boldsymbol{v}_i'$ for all vertices
return $\boldsymbol{V}(t+1) = (\boldsymbol{v}_1(t+1), \cdots, \boldsymbol{v}_m(t+1))$

The overall simulation of cellular pattern formation is carried out by repeatedly applying this algorithm to model different biological phenomena, with pre-defined time-dependent volume changes and cell properties changes assigned as input. Stochasticity and other physical factors can be incorporated in schemes that assign these changes.

Topological Changes of Cellular Pattern

An important ingredient in modeling dynamic changes of cells is an accurate account of all topological changes. We discuss these changes below.

Cell Birth, Cell Division, and Cell Death Topological changes occur during cell birth, cell division, and cell death. In our model, a new cell is generated at cell birth. We model this by inserting a new disk. A new cell is also formed if an existing cell divides. For cell division, we add an edge inside the existing dividing cell.

When a cell dies gradually, its volume decreases, and it eventually disappears, making new empty room in the space. A cell can also disappear suddenly. In both cases of cell death, we carry out two primitive operations. First, the outer edge of the dying cell is removed at the moment when cell dies; Second, the inner edges of all the cells contacting the dying cell are replaced with outer edges.

Cell Contact Changes In addition to cell birth and cell death, there are three additional types of topological changes when cells grow or shrink and their boundaries move, resulting in cell rearrangement. We use three primitives to model these topological changes, which occur when the same space would be occupied by more than one cell:

Edge Insertion When two cells grow, they may come in contact with each other. When this happens, we add an inner edge to represent the newly formed intersection plane.

Void Removal When three cells are grown together, new inner edges are introduced between two contacting cells. At the moment when three cells meet at a common vertex, we

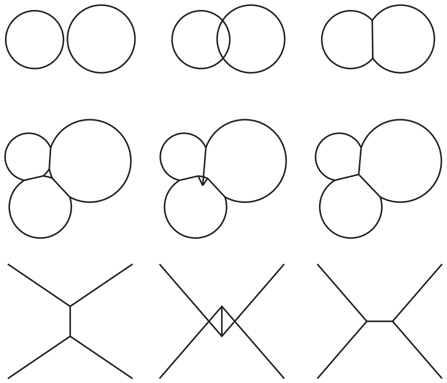

Figure S 5: Possible topological changes when cells grow or when their boundaries move. (top) Edge insertion. When two isolated cells come into contact, we add an edge to represent the intersection plane of the boundary between these two cells. (middle) Void removal. When three cells come into contact, the curve triangular empty space is replaced by a vertex where the inner edges meet. (bottom) Edge flip. When two previously disconnected cells expand to meet while pushing away two previously connected cells, we replace one inner edge with a new inner edge.

need to replace the curved triangular empty space (void) with a new vertex where the three inner edges meet.

Edge Flip When two originally disconnected cells expand and come into contact, they may squeeze away two previously contacting cells. In this case, we remove the inner edge between two cells originally in contact, and add a new inner edge between the two cells that now come into contact.

Together with the three topological changes of inserting a cell due to cell birth, inserting an inner edge due to cell division, and deleting a cell due to cell death, we exhaust all possible topological changes of cellular patterns modeled in two dimensional space. In our model, these topological changes can occur at any discrete time step during the simulation.

References

[1] Sullivan S Jr, Hardy B, Holland C (1964) Formation of air bubbles at orifices submerged beneath liquids. *AIChE J* 10:848–854.

[2] Isenberg C (1992) *The Science of Soap Films and Soap Bubbles.* (Dover).

[3] Weaire D, Hutzler S (1999) *The Physics of Foams.* (Oxford Univ. Press, Oxford, UK).

[4] Hayashi T, Carthew R (2004) Surface mechanics mediate pattern formation in the developing retina. *Nature* 431:647–652.

[5] Sekimura T, Madzvamuse A, Wathen A, Maini P (2000) A model for colour pattern formation in the butterfly wing of papilio dardanus. *Proceedings: Biological Sciences* 267:851–859.

[6] Honda H, Tanemura M, Yoshida A (2000) Differentiation of wing epidermal scale cells in a butterfly under the lateral inhibition model–appearance of large cells in a polygonal pattern. *Acta Biotheor* 48:121–136.

[7] Honda H, Tanemura M, Yoshida A (1990) Estimation of neuroblast numbers in insect neurogenesis using the lateral inhibition hypothesis of cell differentiation. *Development* 110:1349–1352.

[8] Madzvamuse A, Maini P, Wathen A, Sekimura T (2002) A predictive model for color pattern formation in the butterfly wing of papilio dardanus. *Math J* 32:325–336.

[9] Mizushima-Sugano J, Maeda T, Miki-Noumura T (1983) Flexural rigidity of singlet microtubules estimated from statistical analysis of their contour lengths and end-to-end distances. *Biochim Biophys Acta* 755:257–262.

[10] Burnside B (1971) Microtubules and microfilaments in newt neuralation. *Dev Biol* 26:416–441.

[11] Madreperla S, Adler R (1989) Opposing microtubule- and actin-dependent forces in the development and maintenance of structural polarity in retinal photoreceptors. *Dev Biol* 131:149–160.

[12] Brown M, Hallam J, Colucci-Guyon E, Shaw S (2001) Rigidity of circulating lymphocytes is primarily conferred by vimentin intermediate filaments. *J Immunol* 166:6640–6646.

[13] Hotani H, Miyamoto H (1990) Dynamic features of microtubules as visualized by dark-field microscopy. *Adv Biophys* 26:135–156.

[14] Domnina L, Rovensky J, Vasiliev J, Gelfand I (1985) Effect of microtubule-destroying drugs on the spreading and shape of cultured epithelial cells. *J Cell Sci* 74:267–282.

[15] Harris A, Wild P, Stopak D (1980) Silicone rubber substrata: a new wrinkle in the study of cell locomotion. *Science* 208:177–179.

[16] Hilbert D, Cohn-Vossen S (1922) *Geometry and the Imagination* (Chelsea).

[17] Snyman J (2005) *Practical Mathematical Optimization: An Introduction to Basic Optimization Theory and Classical and New Gradient-Based Algorithms* (Springer Publishing).
